# Thoracic Surgery

**Published:** 1905-11-18

**Authors:** 


### THORACIC SURGERY.

**Empyema.**—Whilst the chief difficulty in the radical cure of empyema is met with in dealing with chronic cases, yet there are several important points in the treatment of acute empyema the observance of which would often prevent the case from becoming chronic. In its early stages the pus cavity in the chest is only lined by a thin layer of lymph, and if the suppuration can be stopped before this layer of lymph has become organised to form a thick mass of fibrous tissue, the cavity will rapidly close. Berry [1] shows that there are three main principles to be observed in the surgical treatment of acute empyema. (1) The condition should be thoroughly treated directly it is recognised, because the longer it is allowed to progress the greater is the retraction of the lung, and the thicker will be the layer of lymph lining the cavity. And as mere aspiration is nearly always ineffectual, no time should be wasted in trying this method, except as an immediate preliminary to the more radical method. (2) The opening should be made in a position in which rapid and effectual drainage can take place, that is at the ninth rib, in a line with the angle of the scapula behind. In order to do this the patient may be held lying on his back, so that the affected side projects over the edge of the table. But this method demands one or two extra assistants, and the surgeon's position is rather an awkward one. And if the patient is placed almost on his face, lying on the affected side, these difficulties will be avoided, and there will be no fear of the opening not being far enough back. (3) The opening must be large enough for free drainage. This will necessitate removing a piece of rib—preferably the ninth—about two inches long. The intercostal spaces which may be large and bulging before the pus is evacuated, become much contracted afterwards, and the adjoining ribs occlude the tube by pressure. Anæsthesia for this operation should be of the lightest character, either by local anæsthesia or by a few whiffs of chloroform only, and the actual operation should not last more than five to ten minutes. When, however, there is a chronic empyema, the method of treatment is a matter on which there is some difference of opinion. In estimating the direction and extent of such a cavity most surgeons are content to use a bent probe, but owing to the obliquity of the sinus leading into the pleura, and to bands of adhesion, this is not always satisfactory, and Grünbaum [2] has suggested a method by which the volume of the cavity can be accurately measured. He fits a rubber tracheotomy tube by plaster into the sinus in the patient's chest, after the cavity has been emptied. The rubber tube is then connected to one tube of a Woulff's bottle, the other tube of the bottle being fitted with a three-way piece, which connects it with an exhaust pump and manometer. The pump has a known volume, and the fall of pressure shown by the manometer after one suction of the pump will give data from which the volume of the cavity in the patient's chest can be calculated. The following method dispenses with the calculation. The apparatus is fixed to a large bottle, partly filled with water, after the above observation has been recorded, and the pump is again withdrawn once. If the manometer shows a less fall in pressure, water is added to the bottle until the fall in pressure is the same as when the apparatus was attached to the chest. Then the volume of air in the bottle is the same as that in the empyema cavity. Berry [1] expresses the opinion of most English surgeons when he says that an efficiently performed Estländer's operation is all that is wanted to cure a chronic empyema. But it is not sufficiently recognised that efficiency in this operation involves two things—namely, the removal of portions of a sufficient number of ribs, generally from the second to the ninth inclusive, and the removal of a sufficiently long piece of each rib, from 3 to 6 inches. Pringle [3] considers that thoracoplastic operations would seldom be necessary if, when the empyema is first opened, the adhesions of the lung were systematically separated. This is done by the finger, beginning behind, then working down to the diaphragm, and lastly forwards and upwards, enlarging the opening, if necessary, by removing a piece of a second rib and sometimes employing a thick metal bougie or periosteal elevator. When, in spite of this, thoracoplasty is required at a later date, this author considers that the ordinary Estländer's operation is often not effectual, and if it is effectual it produces great scoliosis. He therefore recommends that two portions of each rib, from the second to the ninth, should be removed, the one in front of the anterior axillary line, and the other as far back as possible; and, in addition to this, the whole of the sixth and ninth ribs, and sometimes the third, can be taken away. This allows the side of the thorax to fall right in on the lung without causing scoliosis. If the case be one of long standing the parietal and visceral pleura will each be very thick and firm, and under these circumstances Pringle removes the portions of parietal pleura underlying the parts of the ribs he has removed (Schede's operation), and decorticates the lung (Delorme's operation). But, as Berry [1] points out, this is a procedure which has a mortality of 15 per cent. to 20 per cent., and is scarcely justified when the more simple methods of thoracoplasty give such good results. But there are always certain cases, intermediate between the acute and the chronic, in which there is a doubt whether the cavity will close without a plastic operation, and it has been suggested by Grünbaum [2] and Woods [4] independently, that by applying a suction apparatus to the sinus, so as to assist the expansion of the lung, for one or two hours daily, the cavity may be made to fill rapidly. Woods attaches a large air bottle to the chest by a tube which has an air-tight pneumatic pad fixing it into the sinus. The bottle is partly exhausted by a pump. Grünbaum uses a more complicated pumping arrangement, but the principle involved is the same. Mathews [5] relates a case of localised empyema in a youth of 19, when two cavities were opened and successfully drained on different dates. The first cavity contained staphylococci and a leptothrix, while in the second the leptothrix only could be found. The pus was thick and foul smelling, and showed the presence of numerous small yellow granules of firm consistence of the size of a pin's head. These granules consisted almost entirely of felted masses of leptothrix threads. The filaments stained readily, but showed some clubbing. The leptothrix racemosa is constantly present in the mouth, and it is noteworthy that this patient had had eight teeth removed (at the base of one of which was an alveolar abscess) three weeks before the chest symptoms began, and it is possible that he may have inhaled some of the carious debris during the extraction, and that the leptothrix may have thus gained entrance.

[1] Clin. Jour., May 3, 1905. [2] Brit. Med. Jour., April 15, 1905. [3] Ibid. [4] Ibid., May 20, 1905. [5] Pract., Feb., 1905.

